# *VARS2* and *TARS2* Mutations in Patients with Mitochondrial Encephalomyopathies

**DOI:** 10.1002/humu.22590

**Published:** 2014-05-14

**Authors:** Daria Diodato, Laura Melchionda, Tobias B Haack, Cristina Dallabona, Enrico Baruffini, Claudia Donnini, Tiziana Granata, Francesca Ragona, Paolo Balestri, Maria Margollicci, Eleonora Lamantea, Alessia Nasca, Christopher A Powell, Michal Minczuk, Tim M Strom, Thomas Meitinger, Holger Prokisch, Costanza Lamperti, Massimo Zeviani, Daniele Ghezzi

**Affiliations:** 1 Unit of Molecular Neurogenetics, Fondazione IRCCS (Istituto di Ricovero e Cura a Carattere Scientifico) Istituto Neurologico “Carlo Besta”Milan, Italy; 2 Institute of Human Genetics, Helmholtz Zentrum MünchenNeuherberg, Germany; 3 Institute of Human Genetics, Technische Universitat MünchenMunich, Germany; 4 Department of Life Sciences, University of ParmaParma, Italy; 5 Unit of Child Neurology, Fondazione IRCCS Istituto Neurologico “Carlo Besta”Milan, Italy; 6 Department of Pediatrics, University of SienaSiena, Italy; 7 MRC Mitochondrial Biology UnitCambridge, United Kingdom

**Keywords:** aminoacyl tRNA syntethases, mitochondrial disease, OXPHOS defect, encephalomyopathy, *VARS2*, *TARS2*

## Abstract

By way of whole-exome sequencing, we identified a homozygous missense mutation in *VARS2* in one subject with microcephaly and epilepsy associated with isolated deficiency of the mitochondrial respiratory chain (MRC) complex I and compound heterozygous mutations in *TARS2* in two siblings presenting with axial hypotonia and severe psychomotor delay associated with multiple MRC defects. The nucleotide variants segregated within the families, were absent in Single Nucleotide Polymorphism (SNP) databases and are predicted to be deleterious. The amount of VARS2 and TARS2 proteins and valyl-tRNA and threonyl-tRNA levels were decreased in samples of afflicted patients according to the genetic defect. Expression of the corresponding wild-type transcripts in immortalized mutant fibroblasts rescued the biochemical impairment of mitochondrial respiration and yeast modeling of the *VARS2* mutation confirmed its pathogenic role. Taken together, these data demonstrate the role of the identified mutations for these mitochondriopathies. Our study reports the first mutations in the *VARS2* and *TARS2* genes, which encode two mitochondrial aminoacyl-tRNA synthetases, as causes of clinically distinct, early-onset mitochondrial encephalopathies.

## Introduction

Mitochondrial disorders include widely heterogeneous clinical syndromes, frequently presenting as encephalo- and/or cardiomyopathies, associated with a broad range of causative genes [Ghezzi and Zeviani, 2012]. Their biochemical signature is the presence of defective activity in the mitochondrial respiratory chain (MRC) complexes, resulting in faulty oxidative phosphorylation (OXPHOS), which can impair ATP production. Mutations in several genes associated with defects of mitochondrial protein synthesis, affecting either mitochondrial DNA (mtDNA) or nucleus-encoded genes, have been reported in a range of mitochondrial syndromes [Rotig, 2011].

Translation of the 13 mtDNA-encoded, OXPHOS-related proteins takes place within the mitochondrial matrix. This complex process requires ribosomal and transfer RNAs (rRNAs and tRNAs), encoded by mtDNA, and more than 100 proteins, encoded by nuclear genes, translated by cytosolic ribosomes and imported into mitochondria. These include aminoacyl-tRNA synthetases (aaRSs), ribosomal proteins, ribosomal assembly factors, tRNA- and rRNA-modifying enzymes, initiation, elongation, and termination factors [Smits et al., 2010]. Mutations in any component of the mitochondrial translation machinery can in principle cause inherited mitochondrial disorders affecting the MRC complexes containing mtDNA-encoded subunits (cI, cIII, cIV, cV), with the preservation of complex II, the only complex which has no mtDNA-encoded proteins.

An increasing number of mitochondrial translation disorders are caused by mutations in genes encoding mt-aaRSs [Konovalova and Tyynismaa, 2013], which catalyze the ligation of specific amino acids to their cognate tRNAs, a crucial process for faithful protein synthesis. Mitochondrial and cytoplasmic aaRSs are encoded by distinct nuclear genes, with the exception of GARS and KARS, which are present in both cellular compartments. The term *aaRS2* indicates the gene coding for the mitochondrial enzyme. Mutations in *aaRS2* genes have been associated with diverse clinical presentations, usually characterized by early-onset and autosomal recessive transmission. A relatively tight genotype–phenotype correlation has been reported for most of these syndromes, although the basis of cell- or tissue-specific damage remains unclear, since all mt-aaRSs are ubiquitous enzymes operating in the same pathway [Rotig, 2011].

We report here the identification by whole-exome sequencing (WES) of the first described mutations in *VARS2* (MIM# 612802) and *TARS2* (MIM# 612805) in patients with clinical presentations compatible with mitochondrial disorders and OXPHOS deficiency. All identified variants were not present in SNPs databases, predicted to be deleterious, segregated within the families, and associated with decreased aminoacylation of the corresponding tRNAs in mutant immortalized fibroblasts; moreover, their pathogenic role was proven by using complementation assays on immortalized fibroblasts from patients and, for the *VARS2* mutation, on a specific recombinant yeast model.

## Patients and Methods

Informed consent, approved by the Ethical Committee of the Foundation IRCCS Istituto Neurologico “C.Besta”, Milan, Italy, in agreement with the Declaration of Helsinki, was signed by the parents of the patients.

### Patient 1 (P1)

P1 is an 8-year-old patient, born at the 37th week of gestation by normal delivery. Early after birth, he showed psychomotor delay, facial dysmorphisms, and microcephaly. He never achieved autonomous ambulation. At 4 years of age, onset of partial seizures was characterized by version of the head towards right, followed by clonic and tonic movements initially involving the right arm and subsequently the left one. Between the episodes of focal seizures, bilateral asynchronous myoclonic jerks, in the form of epilepsia partialis continua, involved both upper limbs. On several occasions, seizures evolved into status epilepticus.

**Figure 1:**
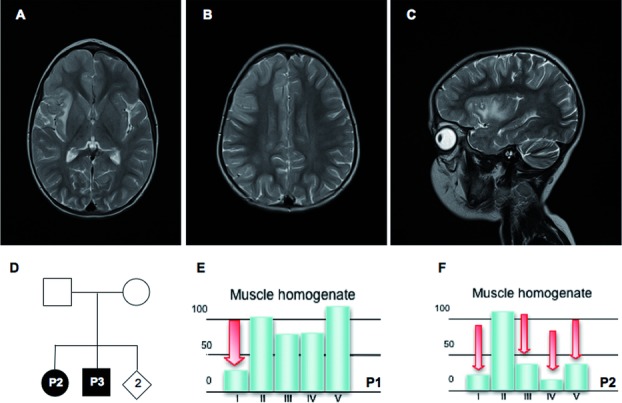
Patients’ phenotypical, biochemical, and mutational features. **A–C**: Axial (**A**, **B**) and sagittal (**C**) T2-weighted images of patient 1, taken at 4 years of age. **A**: Note T2-hyperintense lesions in the frontotemporal right cortex. There are also signal abnormalities in the semioval center (**B**) and the insula (**C**). **D**: Pedigree of patients 2 and 3. Black symbols designate affected subjects. **E**: Bar graphs with MRC complex activities in P1 muscle homogenate. I, II, III, IV, and V correspond to the activities of complexes I, II, III, IV, and V, respectively, normalized for the citrate synthase activity. **F**: Bar graphs with MRC complex activities in P2 muscle homogenate. I, II, III, IV, and V correspond to the activities of complexes I, II, III, IV, and V, respectively, normalized for the citrate synthase activity.

Brain MRI displayed hyperintense lesions in the periventricular regions, the insulae, and the frontotemporal right cortex (Fig. 1A–C). Brain proton magnetic resonance spectroscopy revealed a lactate peak in the frontal white matter. EEG showed slow background activity and epileptic abnormalities in the frontotemporal and occipital derivations of both sides (R > L). Muscle computerized tomography showed bilateral quadriceps hypotrophy but no evident histological alteration was observed in a muscle biopsy.

### Patients 2 and 3 (P2, P3)

P2 and P3 were two siblings (Fig. 1D) presenting with axial hypotonia and limb hypertonia, psychomotor delay, and high levels of blood lactate. They both died a few months after birth of a metabolic crisis.

The brain MRI of P2 at 5 months of age showed a thin corpus callosum and hyperintense lesions of the globi pallidi (images not available); no clear alterations were present in the MRI of P3, taken at 3 months of age. Autoptic examination was carried out on P2 and revealed subsarcolemmal lipofuscin-positive deposits at the trichrome Gomori staining of muscle, cerebral spongiosis, and hepatic steatosis.

### Biochemical Studies

Biochemical measurement of individual OXPHOS complex activities was performed by standard spectrophotometric assays [Bugiani et al., 2004] in muscle homogenate and digitonin-treated skin fibroblasts. Oxygen consumption rate was measured using a SeaHorse FX-96 apparatus (Bioscience, Copenhagen, Denmark) [Invernizzi et al., 2012] in fibroblasts grown either in glucose-rich or in 5 mM galactose, glucose-free DMEM medium for 72 h.

### Molecular Analysis

Total genomic DNA was extracted by standard methods from peripheral blood lymphocytes or muscle biopsies. Southern blot analysis of muscle mtDNA and sequencing of the entire mtDNA was performed as described [He et al., 2002]. WES and variant filtering were performed as previously reported [Haack et al., 2012]. Exons and exon–intron boundaries of human *VARS2* (NM_001167734.1; NP_001161206.1) and *TARS2* (NM_025150.4; NP_079426.2) were amplified using primers listed in Supp. Table S1, and analyzed by Sanger sequencing. Nucleotide numbering reflects cDNA numbering with +1 corresponding to the A of the ATG translation initiation codon in the reference sequence, as indicated in the guidelines of this journal (http://www.hgvs.org/mutnomen). The initiation codon is codon 1. All variants reported have been submitted to LSDB (http://www.lovd.nl/VARS2; http://www.lovd.nl/TARS2). Total RNA was isolated from cell pellets using the RNeasy Mini Kit (Qiagen, Milan, Italy) and reverse transcribed to cDNA using the GoTaq® 2-Step RT-qPCR System (Promega, Madison, WI), following manufacturer's recommendations. *VARS2* and *TARS2* expression in DNase-treated cDNA samples was determined using reverse transcription quantitative PCR with specific amplicons and SYBR-green chemistry (Supp. Table S1).

### Western Blot Analysis

Approximately 10^6^ cells from patients and controls were trypsinized, pelleted, sonicated, and solubilized, as described elsewhere [Tiranti et al., 1999]. SDS-polyacrylamide gel of 50 μg protein/lane and Western blot analysis were performed using antibodies against VARS2 (Mitosciences, Eugene, OR), TARS2 (GeneTex, Irvine, CA), and HSP60 (Abcam).

### tRNA Aminoacylation Assay

Total RNA was extracted from fibroblasts using Trizol reagent (Life Technologies, Carlsbad, CA) following the manufacturer's instructions, with the final pellet resuspended in 10 mM NaOAc at pH 5.0 and kept at 4°C to preserve the aminoacylation state. For the deacylated control, the pellet was resuspended in 200 mM Tris-HCl at pH 9.5 and incubated at 75°C for 5 min, followed by RNA precipitation and resuspension in 10 mM NaOAc at pH 5. Fifteen micrograms of each RNA sample was separated by acid-urea PAGE as described previously [Köhrer and RajBhandary, 2008] and electroblotted to a nylon membrane at 400 mA for >6 h in 40 mM Tris-HCl, pH 8.0, and 2 mM EDTA. Following UV cross-linking (0.120 J), the membrane was hybridized with appropriate radiolabeled riboprobes [Minczuk et al., 2011]. Northern blots were quantified by PhosphorImager analysis with ImageQuant software (Molecular Dynamics, Sunnyvale, CA).

### Lentiviral Transduction

The wild-type (wt) cDNAs from *VARS2* or *TARS2* were cloned into the pLenti6.3/V5-TOPO Vector (Life Technologies), and virions were obtained as previously described [Zhang et al., 2009]. Fibroblasts were immortalized with pRNS-1 by a lipofectin transfection [Litzkas et al., 1984]. Mutant and wt immortalized fibroblasts were infected with viral supernatant and selected upon exposure to 2 μg/ml Blasticidin (Life Technologies).

### Yeast Studies

A detailed description of culture media, plasmids, and strains used for yeast studies is reported in the Supp. Methods, with primers listed in Supp. Table S2. Yeast growth, respiratory activity, and in vitro mtDNA protein synthesis were performed as previously described [Barrientos et al., 2009; Goffrini et al., 2009]. Division time and respiration were measured after growth in SD medium without valine or with valine 40 μg/ml, while mtDNA protein synthesis was measured after growth on SC medium supplemented with 2% galactose and 0.2% glucose.

## Results

### Biochemical Findings

Biochemical assays of MRC complex activities revealed an isolated complex I deficiency (25% of residual activity) in muscle homogenate of P1 (Fig. 1E), but no enzymatic defects in fibroblasts. However, oxygen consumption performed in P1 fibroblasts cultured in either glucose or galactose medium showed defective respiration rate compared to control (Supp. Fig. S1).

Biochemical analysis of P2 showed multiple deficiency of the MRC complex activities in muscle homogenate (Fig. 1F), whereas no defect was observed in fibroblasts; P3 muscle showed the same multiple defect, whereas the MRC complex activities were all normal in a postmortem liver specimen. Oxygen consumption rate of P2 fibroblasts was in the control range in glucose medium, but lower than normal in galactose medium, compared to control cells (Supp. Fig. S1). No fibroblasts were available from P3.

### Mutation Detection

In all patients, mtDNA sequencing revealed no pathogenic mutations and Southern blot analysis was negative for mtDNA deletions or depletion. We then looked for mutations in nuclear genes by WES, performed on genomic DNA from P1 and P3. After filtering to exclude common SNPs (>0.1%), the remaining nonsynonymous/splice site (NS/SS) changes and microinsertions/deletions (INS/DEL) were prioritized according to the presence of homozygous or compound heterozygous mutations, as expected for recessive transmission suggested by the structure of our pedigrees, and for known or predicted mitochondrial localization of the corresponding protein [Elnster et al., 2008; Haack et al., 2012; Ghezzi et al., 2012].

**Figure 2:**
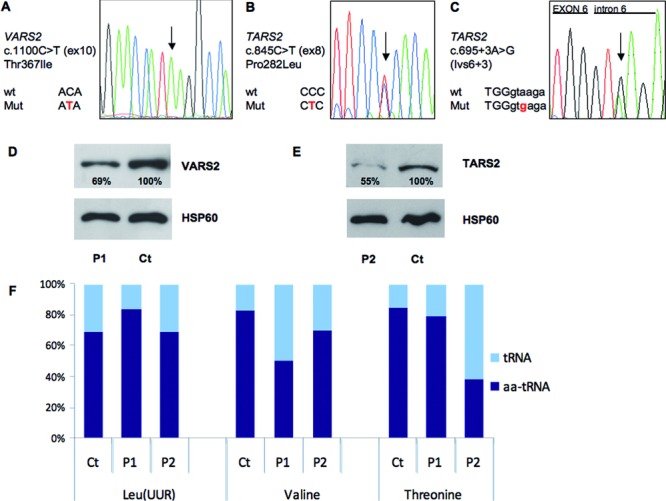
Molecular and protein characterization of *VARS2* and *TARS2* mutations. **A**: Electropherogram of the *VARS2* genomic region encompassing the c.1100C>T nucleotide substitution in patient 1. **B–C**: Electropherograms of the *TARS2* genomic regions encompassing the c.845C>T (**B**) and c.695+3A>G (**C**) nucleotide substitutions in patient 3. **D**: Western Blot analysis of fibroblasts from patient 1 (P1) and control fibroblasts (Ct) using antibodies against VARS2 and HSP60; the latter was taken as a loading control. The reported percentages correspond to the values of VARS2/HSP60 signals obtained by densitometric analysis in three independent experiments. **E**: Western Blot analysis of fibroblasts from patient 2 (P2) and control fibroblasts (Ct) using antibodies against TARS2 and HSP60 proteins; the latter was taken as a loading control. The reported percentages correspond to the values of TARS2/HSP60 signals obtained by densitometric analysis in three independent experiments. **F**: Aminoacylation levels of mitochondrial tRNA^Val^, tRNA^Thr^, and tRNA^LeuUUR^ in control, patient 1 (P1), and patient 2 (P2) fibroblasts measured by quantification of Northern blots (Supp. Fig. S4) performed as described in the Patients and Methods section.

In P1, the filtration strategy revealed the presence of a homozygous missense mutation (c.1100C>T, p.Thr367Ile, Fig. 2A) in *VARS2*, the gene encoding the mitochondrial valyl tRNA-synthetase. Thr367 is conserved in several species, including yeast (Supp. Fig. S2), and the change Thr367Ile has high scores for pathogenicity according to different bioinformatic tools (Supp. Table S3). The mutation was present in heterozygous state in both parents.

Following identical procedures for P3 analysis, we identified two variants in *TARS2*, encoding the mitochondrial threonyl tRNA-synthetase: a missense mutation (c.845C>T, p.Pro282Leu) and a nucleotide change in position +3 of intron 6 (g.4255A>G; c.695+3A>G) (Fig. 2B–C). Both changes were present also in his affected sister P2, and are predicted to be deleterious (Supp. Table S3). Father and mother were heterozygous carriers for the SS and the missense mutation, respectively; one healthy sibling was heterozygous for the SS mutation, while the other was negative for both. Pro282 is conserved in mammals and bird, and present in the yeast Ths1 (Supp. Fig. S2), corresponding to the cytosolic, and possibly also to a mitochondrial, threonyl-tRNA synthetase. All the identified nucleotide substitutions were not reported in public databases, including dbSNPs and NHLBI Exome Sequencing Project.

### Analysis of *VARS2* and *TARS2* Transcripts, Protein Levels, and Aminoacylation of Cognate Mitochondrial tRNAs

As expected, the homozygous missense mutation did not alter the *VARS2* transcript levels in P1 fibroblasts (Supp. Fig. S3). However, the amount of the VARS2 protein detected by Western blot analysis was moderately decreased compared to control fibroblasts, suggesting partial instability of the mutant protein (Fig. 2E).

In order to evaluate the effect of the intronic variant on *TARS2* transcript, we amplified cDNAs extracted from P2 and control fibroblasts. In P2, we did not detect any aberrant species but the band corresponding to the full-length transcript was found to contain only the mutated nucleotide T in position c.845, suggesting that the intronic mutation in the SS determines an aberrant and highly unstable *TARS2* transcript (Supp. Fig. S3). Quantitative real-time PCR analysis revealed diminished expression of *TARS2* (Supp. Fig. S3) and Western Blot analysis showed a clear decrease in TARS2 protein in P2 fibroblasts compared to controls, confirming the hypothesis on the null contribution of the allele with the intronic mutation (Fig. 2F).

Next, we measured the levels of aminoacylated tRNAs (threonyl-tRNA, valyl-tRNA, and leucyl-tRNA as a control) in P1, P2, and a control fibroblast line. In P1, charged tRNA^Val^ was moderately decreased compared to control fibroblasts. Severe decrease in the aminoacylated tRNA^Thr^ was observed in P2 fibroblasts. No appreciable difference in aminoacylation level was found for leucyl-tRNA between control, P1, and P2 fibroblasts (Fig. 2F; Supp. Fig. S4).

### Yeast Studies

To test the possible deleterious effect of the mutations found in humans, we used a *Saccharomyces cerevisiae* yeast model. However, this investigation was possible only for the *VARS2* but not for the *TARS2* gene. Yeast has two genes encoding threonyl-tRNA synthetases, *THS1* and *MST1*. *THS1* encodes a cytoplasmic isoform and a putative mitochondrial isoform, which aminoacylates only the canonical tRNA^Thr(UGU)^; *MST1* encodes the mitochondrial isoform, which aminoacylates both mt-tRNA^Thr(UGU)^ and the noncanonical mt-tRNAThr^(UAG)^, whose corresponding CUN codon encodes, in yeast but not in humans, threonine instead of leucine [Su et al., 2011; Ling et al., 2012]. The human Pro282 is conserved in Ths1, but not in Mst1, which lacks the N-terminal region containing this amino acid. Introduction of the corresponding mutation in *THS1* would not affect mtDNA-dependent protein synthesis, since the mitochondrial enzyme Mst1 is present and can aminoacylate all the mt-tRNA^Thr^, whereas introduction of mutant (or wt) *THS1* in a *MST1Δ* strain would not allow the mtDNA-dependent protein synthesis since the mt-tRNA^Thr(UAG)^ would not be aminoacylated, and CUN codons would not be translated.

Contrariwise, the mutated Thr367 residue in human *VARS2* is conserved in the yeast ortholog *VAS1* (Thr380). However, *VAS1* codes for both the cytosolic and mitochondrial valyl tRNA-synthetases. We disrupted *VAS1* by homologous recombination and reexpressed the wt cytosolic isoform, thus generating a viable but OXPHOS incompetent strain (*cytvas1*), which lacked the mitochondrial isoform. In this strain, we expressed either the wt *VAS1* gene (*VAS1*), or a Thr380Ile mutant allele (*vas1^T380I^*), equivalent to the human Thr367Ile mutation (Supp. Fig. S2).

**Figure 3:**
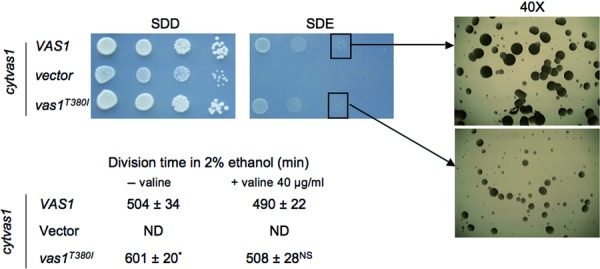
Yeast growth studies. Growth of *cytvas1* strain transformed with wt *VAS1, vas1*^T380I^ mutant allele or empty plasmid on SD medium supplemented with 2% glucose (SDD, left panel) or 2% ethanol (SDE, right panel). Cells were pregrown on SDD and plated after serial dilutions to obtain spots of 5 × 10^4^, 5 × 10^3^, 5 × 10^2^, and 5 × 10^1^ cells/spot. Pictures were taken after 2 days of growth on SDD and 3 days on SDE. Colonies spotted on the third spot of SDE were magnified with a Leica LM 2000 microscope. Division time was calculated by growing cells on liquid SDE medium and measuring the optical density at 600 nm every 2 h when cells were in log phase of growth. Values are mean of three independent experiments. *P < 0.05 by using a two-tailed, paired *t*-test.

The strain expressing *vas1^T380I^* showed a division time in ethanol higher than the strain expressing *VAS1* (Fig. 3), suggesting an OXPHOS-dependent growth defect. Accordingly, the respiration rate in *vas1^T380I^* strain was slightly but significantly lower than in *VAS1* strain (Supp. Fig. S5). No obvious alterations were observed in the in vivo mitochondrial protein synthesis assay (Supp. Fig. S5). Interestingly, the supplementation of valine (40 μg/ml) in the culture medium led to normalization of the division time (Fig. 3) and recovery of respiration for *vas1^T380I^* strain (Supp. Fig. S5), suggesting that Thr380 maps to the substrate binding site and its substitution with Ile380 yields a Km defect.

### Complementation Studies in Fibroblasts

**Figure 4:**
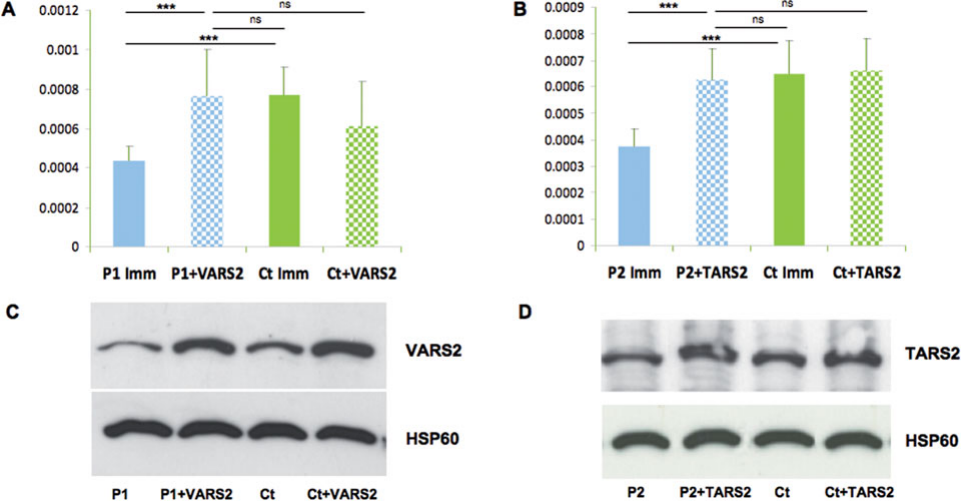
Complementation studies in mutant fibroblasts. **A**: Oxygraphy performed in immortalized P1 and control fibroblasts cultured in galactose medium in naïve condition and after infection with wt cDNA of *VARS2* (+VARS2). Y-axis values correspond to the maximal respiration rate, expressed as pMolesO_2_/min/cell. Data are represented as mean ± SD. Two-tail, paired *t*-test was applied for statistical significance. ***P < 0.001; ns: nonsignificant (*P* > 0.01). **B**: Oxygraphy performed in immortalized P2 and control fibroblasts cultured in galactose medium in naïve condition and after infection with wt cDNA of *TARS2* (+TARS2). Y-axis values correspond to the maximal respiration rate, expressed as pMolesO_2_/min/cells. Data are represented as mean ± SD. Two-tail, paired *t*-test was applied for statistical significance. ***P < 0.001; ns: nonsignificant (*P* > 0.01). **C**: Western blot analysis of samples reported in Figure 4A using antibodies against VARS2 and HSP60, the latter being taken as a loading control. **D**: Western blot analysis of samples reported in Figure 4B using antibodies against TARS2 and HSP60, the latter being taken as a loading control.

To validate the pathogenic role of the identified variants, we sought for the presence of a biochemical readout of mutant fibroblasts by measuring the oxygen consumption rate in cells grown in a galactose-rich, glucose-free medium, a condition that forces cells to depend on mitochondrial respiration rather than glycolysis for ATP production. To avoid discrepancies in the measurements, due to culture passages of primary fibroblasts, we used immortalized P1 and P2 cell lines. We found a clear decrease in the maximal respiration rate in both P1 and P2 immortalized cells compared to immortalized control fibroblasts, which increased to normal values after transduction with a recombinant lentiviral construct expressing the wt cDNA of either *VARS2* (for P1, and the corresponding control) or *TARS2* (for P2, and the corresponding control) (Fig. 4A and B). Western blot on P1 and P2 fibroblasts after transduction showed an increase in the amount of VARS2 and TARS2 proteins, respectively (Fig. 4C and D).

These results confirm the causative role for *VARS2* and *TARS2* variants in defective mitochondrial respiration of P1 and P2 cells, respectively.

## Discussion

Mutations in genes coding for mitochondrial aminoacyl-tRNA synthetases have been associated with diverse clinical presentations, usually inherited as early-onset autosomal recessive traits [Konovalova and Tyynismaa, 2013]. We and others have reported a strict genotype–phenotype correlation for most of these conditions [Edvardson et al. 2007; Scheper et al., 2007; Sasarman et al. 2012; Steenweg et al., 2012]. Since these genetic defects involve ubiquitously expressed enzymes all engaged in the very same enzymatic step of mitochondrial translation, the mechanisms leading to such different cellular and tissue-specific phenotypes remain unexplained. Different hypotheses have been proposed to elucidate the clinical variability associated with different *aaRS2* mutations, but none seems to explain all aspects of this phenomenon that is probably the result of several mechanisms altered in *aaRS2* mutant patients [Konovalova and Tyynismaa, 2013].

However, and similar to our *VARS2* and *TARS2* mutations, most mutations in *aaRS2* genes have been reported in single, or in just a few, cases, which hampers the establishment of definitive genotype to phenotype correlation. For instance, *AARS2* mutations were initially described as associated with hypertrophic cardiomyopathy in two families, but were later found also in patients with leukoencephalopathy and no heart involvement [Dallabona et al., 2014]. Nevertheless, encephalopathy is the most common presentation in *aaRS2* mutations, as in several other OXPHOS disorders, probably because of the high energy request by the central nervous system. For instance, mutations in *DARS2* (MIM# 610956) (mitochondrial aspartyl-tRNA synthetase) have been identified as a recurrent cause of leukoencephalopathy with brainstem, spinal cord involvement, and lactate elevation, whereas *EARS2* mutations (MIM# 612799) (mitochondrial glutamyl-tRNA synthetase) are associated with a leukoencephalopathy with thalamus and brainstem involvement and high lactate. Pontocerebellar hypoplasia 6, a severe infantile encephalopathy with cerebral atrophy and multiple OXPHOS deficiency, is associated with *RARS2* (MIM# 611524) (mitochondrial arginyl-tRNA synthetase) mutations. Complex rearrangements in *MARS2* (mitochondrial methionyl-tRNA synthetase) were described in a cohort of patients with autosomal recessive spastic ataxia with leukoencephalopathy, whereas mutations in *FARS2* (MIM# 611592) (Phenylalanyl-tRNA synthetase) were identified in three patients with fatal epileptic mitochondrial encephalopathy, consistent with Alpers’ syndrome. Interestingly, specific MRI patterns seem to be associated with some *aaRS2* (e.g., *DARS2*, *EARS2*, *RARS2*) mutant patients. In our cases, P1 MRI showed hyperintense lesions involving mainly the insulae and frontotemporal right cortex, whereas P2 MRI was reported to show thinning of corpus callosum (a neuroimaging finding common to several neurodegenerative disorders) and bilateral lesions of the globi pallidi. However, the MRI of P3, brother of P2, was normal, although this can be due to the very early age at which this exam was performed. Additional *VARS2* and *TARS2* cases will clarify whether these MRI patterns are specific or not.

Mutations in *aaRS2*, impairing the translation of the 13 mtDNA-encoded MRC subunits, should in principle be associated with a biochemical deficiency of all MRC complexes, except cII. This biochemical profile was indeed found in muscle samples from the two *TARS2* mutant subjects (P2 and P3), whereas the *VARS2* mutant muscle (P1) showed isolated cI deficiency. Complex I seems to be particularly prone to damage in mtDNA translation defects, as already reported for *MARS2* in patients’ cells and in the corresponding mutant fly model [Bayat et al., 2012] or for mutations in other proteins involved in mitochondrial protein synthesis, such as mitochondrial methionyl-tRNA-formyltransferase [Haack et al., 2012]. This propensity can partly be explained by the fact that seven of the 13 mtDNA-encoded proteins are subunits of cI, and by the complexity of its functional and structural interactions. In patients’ fibroblasts, biochemical assays of individual MRC complexes showed inconsistent alterations, indicating partial preservation of functional proficiency of the mutant enzyme or reflecting the (partial) dispensability from OXPHOS of this cell type. The same biochemical discrepancy has also been reported for mutations in other mt-aaRSs [Gotz et al., 2011; Bayat et al., 2012; Sasarman et al., 2012]. However, we observed a consistent biochemical phenotype in both naïve and immortalized mutant fibroblasts by measuring whole mitochondrial respiration, especially in culturing conditions that force cells to rely on OXPHOS to produce energy. As already reported for *EARS2* mutant fibroblasts, a diminished oxygen consumption rate is likely to depend on the cumulative impairment of the entire set of MRC complexes [Steenweg et al., 2012]. We proved the pathogenic role of the mutations identified in *VARS2* and *TARS2* by showing decreased levels of aminoacylation of the cognate mitochondrial tRNAs and by complementing the biochemical defects in patients’ fibroblasts expressing the corresponding wt cDNAs. This evidence was further corroborated for the *VARS2* mutation by concordant results obtained in a recombinant yeast model.

In conclusion, we identified novel mutations in two genes encoding mitochondrial aminoacyl-tRNA synthetases (*VARS2* and *TARS2*), which have not been appreciated previously as causing mitochondrial disease, thus expanding the list of *aaRS2*-associated diseases. We also confirmed the value of WES for the identification of disease-causing genes even in single patients presenting heterogeneous clinical syndromes but with a biochemically detectable “mitochondrial signature.”

## Supporting Information

Disclaimer: Supplementary materials have been peer-reviewed but not copyedited.

Supplementary information Fig. S1 Respiration in fibroblasts. Fig. S2 Phylogenetic conservation of VARS2 and TARS2. Fig. S3 Analysis of *VARS2* and *TARS2* transcripts. Fig. S4 Aminoacylation of mitochondrial tRNAs. Fig. S5 Yeast studies. Table S1. Primers used for *VARS2* and *TARS2* PCR amplification. Table S2. Oligonucleotides used for the yeast manipulation. Table S3. In silico prediction of pathogenicity for the identified *VARS2* and *TARS2* mutations.

## References

[b1] Barrientos A, Fontanesi F, Diaz F (2009). Evaluation of the mitochondrial respiratory chain and oxidative phosphorylation system using polarography and spectrophotometric enzyme assays. Curr Protoc Hum Genet.

[b2] Bayat V, Thiffault I, Jaiswal M, Tétreault M, Donti T, Sasarman F, Bernard G, Demers-Lamarche J, Dicaire MJ, Mathieu J, Vanasse M, Bouchard JP (2012). Mutations in the mitochondrial methionyl-tRNA synthetase cause a neurodegenerative phenotype in flies and a recessive ataxia (ARSAL) in humans. PLoS Biol.

[b3] Bugiani M, Invernizzi F, Alberio S, Briem E, Lamantea E, Carrara F, Moroni I, Farina L, Spada M, Donati MA, Uziel G, Zeviani M (2004). Clinical and molecular findings in children with complex I deficiency. Biochim Biophys Acta.

[b4] DallaBona C, Diodato D, Kevelam SH, Haack TB, Wong LJ, Salomons GS, Baruffini E, Melchionda L, Mariotti C, Strom TM, Meitinger T, Prokisch H (2014). Novel (ovario)leukodystrophy related to AARS2 mutations. Neurology.

[b5] Edvardson S, Shaag A, Kolesnikova O, Gomori JM, Tarassov I, Einbinder T, Saada E, Elpeleg O (2007). Deleterious mutation in the mitochondrial arginyl-transfer RNA synthetase gene is associated with pontocerebellar hypoplasia. Am J Hum Genet.

[b6] Elstner M, Andreoli C, Ahting U, Tetko I, Klopstock T, Meitinger T, Prokisch H (2008). MitoP2: an integrative tool for the analysis of the mitochondrial proteome. Mol Biotechnol.

[b7] Ghezzi D, Baruffini E, Haack TB, Invernizzi F, Melchionda L, Dallabona C, Strom TM, Parini R, Burlina AB, Meitinger T, Prokisch H, Ferrero I (2012). Mutations of the mitochondrial-tRNA modifier MTO1 cause hypertrophic cardiomyopathy and lactic acidosis. Am J Hum Genet.

[b8] Ghezzi D, Zeviani M (2012). Assembly factors of human mitochondrial respiratory chain complexes: physiology and pathophysiology. Adv Exp Med Biol.

[b9] Goffrini P, Ercolino T, Panizza E, Giachè V, Cavone L, Chiarugi A, Dima V, Ferrero I, Mannelli M (2009). Functional study in a yeast model of a novel succinate dehydrogenase subunit B gene germline missense mutation (C191Y) diagnosed in a patient affected by a glomus tumor. Hum Mol Genet.

[b10] Gotz A, Tyynismaa H, Euro L, Ellonen P, Hyotylainen T, Ojala T, Hamalainen RH, Tommiska J, Raivio T, Oresic M, Karikoski R, Tammela O (2011). Exome sequencing identifies mitochondrial alanyl-tRNA synthetase mutations in infantile mitochondrial cardiomyopathy. Am J Hum Genet.

[b11] Haack TB, Haberberger B, Frisch EM, Wieland T, Iuso A, Gorza M, Strecker V, Graf E, Mayr JA, Herberg U, Hennermann JB, Klopstock T (2012). Molecular diagnosis in mitochondrial complex I deficiency using exome sequencing. J Med Genet.

[b12] He L, Chinnery PF, Durham SE, Blakely EL, Wardell TM, Borthwick GM (2002). Detection and quantification of mitochondrial DNA deletions in individual cells by real-time PCR. Nucleic Acids Res.

[b13] Invernizzi F, D'Amato I, Jensen PB, Ravaglia S, Zeviani M, Tiranti V (2012). Microscale oxygraphy reveals OXPHOS impairment in MRC mutant cells. Mitochondrion.

[b14] Köhrer C, RajBhandary UL (2008). The many applications of acid urea polyacrylamide gel electrophoresis to studies of tRNAs and aminoacyl-tRNA synthetases. Methods.

[b15] Konovalova S, Tyynismaa H (2013). Mitochondrial aminoacyl-tRNA synthetases in human disease. Mol Genet Metab.

[b25] Ling J, Peterson KM, Simonović I, Cho C, Söll D, Simonović M (2012). Yeast mitochondrial threonyl-tRNA synthetase recognizes tRNA isoacceptors by distinct mechanisms and promotes CUN codon reassignment. Proc Natl Acad Sci USA.

[b16] Litzkas P, Jha KK, Ozer HL (1984). Efficient transfer of cloned DNA into human diploid cells: protoplast fusion in suspension. Mol Cell Biol.

[b17] Minczuk M, He J, Duch AM, Etterna TJ, Chlebowski A, Dzionek K, Nijtmans LGJ, Huynen MA, Holt IJ (2011). TEFM (c17orf42) is necessary for transcription of human mtDNA. Nucleic Acids Res.

[b18] Rötig A (2011). Human diseases with impaired mitochondrial protein synthesis. Biochim Biophys Acta.

[b19] Sasarman F, Nishimura T, Thiffault I, Shoubridge EA (2012). A novel mutation in YARS2 causes myopathy with lactic acidosis and sideroblastic anemia. Hum Mutat.

[b20] Scheper GC, van der Klok T, van Andel RJ, van Berkel CGM, Sissler M, Smet J, Muravina TI, Serkov SV, Uziel G, Bugiani M, Schiffmann R, Krageloh-Mann I (2007). Mitochondrial aspartyl-tRNA synthetase deficiency causes leukoencephalopathy with brain stem and spinal cord involvement and lactate elevation. Nat Genet.

[b21] Smits P, Smeitink J, van den Heuvel L (2010). Mitochondrial translation and beyond: processes implicated in combined oxidative phosphorylation deficiencies. J Biomed Biotechnol.

[b22] Steenweg ME, Ghezzi D, Haack T, Abbink TEM, Martinelli D, van Berkel CGM, Bley A, Diogo L, Grillo E, Te Water Naude J, Strom TM, Bertini E (2012). Leukoencephalopathy with thalamus and brainstem involvement and high lactate ‘LTBL’ caused by EARS2 mutations. Brain.

[b26] Su D, Lieberman A, Lang BF, Simonovic M, Söll D, Ling J (2011). An unusual tRNA^Thr^ derived from tRNA^His^ reassigns in yeast mitochondria the CUN codons to threonine. Nucleic Acids Res.

[b23] Tiranti V, Galimberti C, Nijtmans L, Bovolenta S, Perini MP, Zeviani M (1999). Characterization of SURF-1 expression and Surf-1p function in normal and disease conditions. Hum Mol Genet.

[b24] Zhang JC, Sun L, Nie QH, Huang CX, Jia ZS, Wang JP, Lian JQ, Li XH, Wang PZ, Zhang Y, Zhuang Y, Sun YT, Bai X (2009). Down-regulation of CXCR4 expression by SDFKDEL in CD34(+) hematopoietic stem cells: an antihuman immunodeficiency virus strategy. J Virol Methods.

