# Supplementary material for: VARS2 and TARS2 Mutations in Patients with Mitochondrial Encephalomyopathies
Source: Hum Mutat. 2014 May 14;35(8):983–9. doi: 10.1002/humu.22590 (PMC4140549; doi:10.1002/humu.22590)
Supplement: Supplementary file 1 [file humu0035-0983-SD1.pdf]

Supp. Methods: Yeast strains, plasmids and media

The W303-1B genotype is *Matα ade2-1 leu2-3, 112 ura3-1 trp1-1 his3-11, 15 can1-100*. Strains were grown in SD synthetic defined medium (0.69% Yeast Nitrogen base without amino acids ForMedium, Norfolk, UK) supplemented with amino acids and bases necessary for complementing the W303-1B strain auxotrophies, or SC synthetic complete medium (0.69% YNB without amino acids ForMedium, Norfolk, UK,, 1gr/L dropout mix (Kaiser et al., 1994) without uracil, tryptophan and, in case, valine). Media were supplemented with various carbon sources (w/v) (Carlo Erba Reagents, Milan, Italy), in liquid phase or after solidification with 20g/L agar (ForMedium, Norfolk, UK).

VAS1 was cloned under its natural promoter by PCR-amplification with oligonucleotides VAS1CFw and VAS1CRv (Supp. Table S2) and cloning of the *Sac*I-*Pst*I-digested *VAS1* fragment in pFL36 (Bonneaud et al., 1991). The plasmid was inserted in W303-1B through the Li-Ac method (Gietz and Schiestl, 2007). Since disruption of *VAS1* is lethal, we performed disruption of the gene in strain W303-1B harboring pFL36*VAS1*. Disruption was performed through one-step gene disruption by PCR-amplification of KanMX4 cassette (Wach et al, 1994) with primers VAS1DFw and VAS1DRv (Supp. Table S2) and transformation of the former strain, thus obtaining W303-1B *vas1Δ*/pFL36VAS1.

VAS1 fragment was subcloned from pFL36 to pFL38 and pFL39 (Bonneaud et al., 1991). All the mutations were introduced through the overlap method (Ho et al., 1989) with appropriate primers (Supp. Table S2). pFL38*VAS1* was mutagenized to obtain an isoform of *VAS1* encoding the cytoplasmic but not the mitochondrial Vas1 isoform, called *cytvas1*, through mutagenesis of codon 1, which is the initiator codon for the mitochondrial isoform, which was changed to the codon for alanine GCG, as previously performed by others (Wang et al., 2003), thus obtaining pFL38*cytvas1*,. pFL39*VAS1* was mutagenized with appropriate primers (Supp. Table S2) to obtain mutant allele pFL39$vas1^{T380I}$.

W303-1B *vas1Δ* pFL36*VAS1* was co-transformed with pFL38*cytvas1*, and pFL39*VAS1* wt or pFL39$vas1^{T380I}$, and then pFL36*VAS1* was lost through plasmid-shuffling, thus obtaining strains encoding a cytoplasmic wt Vas1 isoform and a wt or mutant Vas1 isoform.

Supp. References

Bonneaud N, Ozier-Kalogeropoulos O, Li GY, Labouesse M, Minvielle-Sebastia L, Lacroute F. (1991) A family of low and high copy replicative, integrative and single-stranded S. cerevisiae / E. coli shuttle vectors. Yeast 7: 609–615.

Gietz RD, Schiestl RH. (2007) High-efficiency yeast transformation using the LiAc/SS carrier DNA/PEG method. Nat Protoc 2:31-34.

Ho SN, Hunt HD, Horton RM, Pullen JK, Pease LR. (1989) Site-directed mutagenesis by overlap extension using the polymerase chain reaction. Gene 77:51-59.

Kaiser C, Michaelis S, Mitchell A. (1994) Methods in Yeast Genetics: a Laboratory Course Manual, Cold Spring Harbor Laboratory Press, Cold Spring Harbor, NY.

Wach A, Brachat A, Pöhlmann R, Philippsen P. (19949 New heterologous modules for classical or PCR-based gene disruptions in Saccharomyces cerevisiae.Yeast 10:1793-1808.

Wang CC, Chang KJ, Tang HL, Hsieh CJ, Schimmel P. (2003) Mitochondrial form of a tRNA synthetase can be made bifunctional by manipulating its leader peptide. Biochemistry 42:1646-51.

Supp. Figure S1

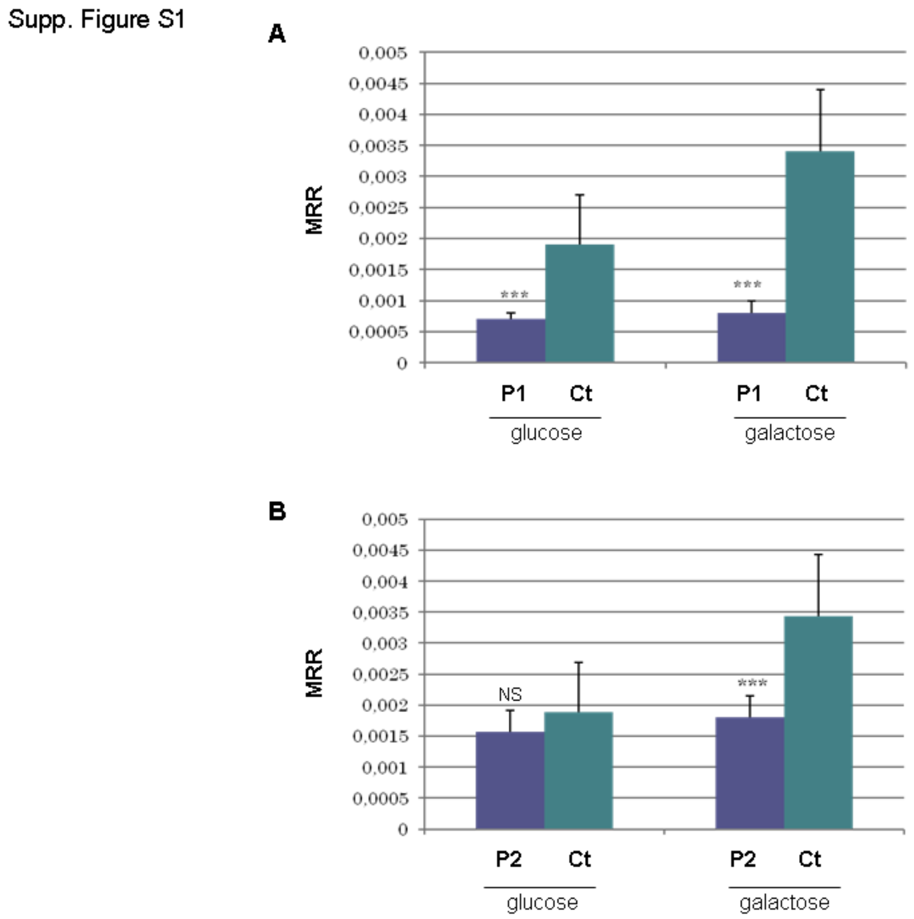

Respiration in fibroblasts

Maximal respiration rate (MRR), measured in fibroblasts from patient 1 (P1, panel A), patient 2 (P2, panel B) and corresponding control subjects (Ct). MRR values are expressed as pMolesO_2/min/cells. Data are represented as mean ± SD. Two-tail, unpaired Student's t test was applied for statistical significance. ***: $p < 0.001$; NS: non-significant ($p > 0.01$).

Supp. Figure S2

```
A.                                 VARS2
Homo_sapiens                      -DAEVVVGTTRPETLPGDVAVAVHPDDSRYTHLHGRQLRHPLMGQPLPLI
407
Mus_musculus                      -DTEIVVGTTRPETLPGDVAVAVHPDDPRYTHLHGRQLRHPLTGQLLPLI
377
Rattus_norvegicus                 -GTEIVVGTTRPETLPGDVAVAVHPDDPRYTHLHGRQLRHPLTGQHLPLI
377
Bos_taurus                        -DAEVVVGTTRPETLPGDVAVAVHPDDSRYTHLHGRQLCHPLTGQLLPLI
457
Monodelphis_domestica             -DTEIVVETTRPETLPGDVAVAVHPDDPRYTHLHGRQLCHPLSGKLLPLI
961
Pseudopodoces_humilis_partial_    -GLELPVATTRPETIFGDVAVAVHPRDPRYLHLQGRQVRHPFSGALLPVV
237
Falco_peregrinus_partial_         -GLELPVATTRPETMLGDVAVAVHPEDPRYTHLHGRQLRHPFTGQLLPVI
382
Anolis_carolinensis               -GEELPVATTRPETMLGDVAVAVHPDDPRYTHLHGKRLCHPFTGQLIPVI
297
Xenopus_tropicalis                -D-EIIVSTTRPETMLGDTAVAVHPNDPRYLRFHGTRLHHPFTGQLIPVV
356
Danio_rerio                       -G-EVAVSTTRPETMLGDVAIAVHPDDPRYKHLHGKRCRHPFTEQLLPIV
363
Apis_mellifera_partial_           ---EIIVATTRPETLFGDVAIAVHPDDERYAKYIGQQVWHTLR-QTYIPI
166
Drosophila_melanogaster           --EEIIVATTRIETMLGDTAVAVHPQDDRYKHLHGKFVVHPFS-TRRLPI
367
Caenorhabditis_elegans            --EEIVVSTTRIETMLGDSGVAVHPDDQRYKHLVGKQCIHPFIPTRNLPI
357
Plasmodium_falciparum             --EKIEIATTRIETMLGDVAVAVHPKDKRYAHLIGKEIVHPFIPNRKIII
320
Trypanosoma_brucei                --DEIIIATTRPETILGDTAVAVHPDDERYKKYHGKRLKCPFRDETIPLI
283
Acanthamoeba_castellanii          --EQIVVSTTRIETMLGDTAIAVHPDDERYKHLHGKFAVHPFNGRRIPII
350
Neurospora_crassa                 --ETIEVATTRPETMLGDTGIAVNPEDPRYTHLVGKFARHPFVDRLLPIV
366
Candida_albicans                  TNEKLTVATTRPETIFGDTAVAVHPKDPRYTHLHGKFVQHPFLDRKLPII
294
Saccharomyces_cerevisiae          --EKLIIATTRPETIFGDTAVAVHPDDDRYKHLHGKFIQHPFLPRKIPII
420
Arabidopsis_thaliana              --GEVIVATTRVETMLGDTAIAIHPDDARYKHLHGKFAVHPFNGRKLPII
407
```

```
B.                                 TARS2
Homo_sapiens                      SGISFPTTELLRVWEAWREEAELRDHRRIGKEQELFFFHELSPGSCFFLP
326
Mus_musculus                      SGISFPKVELLRNWEARREAAELRDHRRIGKEQELFFFHELSPGSCFFLP
331
Rattus_norvegicus                 SGISFPKAELLRNWEARREEAELRDHRRIGKEQELFFFHELSPGSCFFLP
331
Bos_taurus                        SGISFPTAEGLQAWEEWREEAELRDHRRIGKEQELFFFHELSPGSCFFLP
328
Monodelphis_domestica             YGISFPTSEGLRAWEKQREEAAARDHRRIGKDQELFFFHELSPGSCFFLP
325
Gallus_gallus_partial             AAIAFPSTQELQAWQQAQDAAALRDHRRIGKEQELFFFHALSPGSCFFLP
302
Pseudopodoces_humilis_partial     AAVAFPSAQDLEQWERAQHEAAQRDHRRIGKDQELFFFHKFSPGSCFFLP
321
Xenopus_tropicalis                YGISFPDPKMLKEWEKFQEEAKNRDHRKIGREQELFFFHELSPGSCFFLP
345
```

```
Drosophila_melanogaster_isof    YGISFPDPKQLKEWEKLQEEAAKRDHRKIGREQELFFFHELSPGSCFFQP
316
Caenorabditis_elegans_isof      YGISFPDSKQLKEWQKLQEEAAKRDHRKLGKEHDLFFFHQLSPGSAFWYP
346
Trypanosoma_brucei              YGISFPKQTMLTEWKKLQEEAARRDHRTIGRHQQLFHFHEASPGNAFWLP
316
Neurospora_crassa_mitocondrial --------------------PAAP-DHRDLGTQQELFMTSIYSPGSPIFLP
80
Saccharomyces_cerevisiae_Ths1   YGISFPDKKLMDAHLKFLAEASMRDHRKIGKEQELFLFNEMSPGSCFWLP
354
Saccharomyces_cerevisiae_Mst1  --------------------ATPATMTSMVSQRQDLFMTDPLSPGSMFFLP
63
Arabidopsis_thaliana            YGISYPDQKQLKKYLQFLEEAKKYDHRLLGQKQELFFSHQLSPGSYFFLP
341
```

Phylogenetic conservation of VARS2 and TARS2

Aligned protein sequences from different organisms: placental mammals (*H. sapiens*, *M. musculus*, *R. norvegicus*, *B. taurus*), marsupial mammals (*M. domestica*), birds (*G. gallus*, *P. humilis, F. peregrinus*), reptiles (*A. carolinensis*), amphibians (*X. tropicalis*), fishes (*D. rerio*), arthropods (*A. mellifera, D. melanogaster*), worms (*C. elegans*), protozoa (*P. falciparum*, *T. brucei*, *A. castellanii*), fungi (*N. crassa, C. albicans, S. cerevisiae*) and plants (*A. thaliana*).

A. Alignment of VARS2 orthologs. The aminoacid residues corresponding to threonine in position 367 of the human protein, mutated in patient 1, are in red bold.

B. Alignment of TARS2 orthologs. The aminoacid residues corresponding to proline in position 282 of the human protein, mutated in patients 2 and 3, are in red bold. No sequences were reported in databases for reptiles and fishes. *S. cerevisiae* possesses two genes: *MST1* encodes for the mitochondrial isoform, which is able to aminoacylates both the canonical $tRNA^{Thr}$ (codons ACN) and the *Saccharomycetaceae* mitochondrial-specific $tRNA^{Thr}$ (codons CUN); *THS1* encodes for the cytoplasmic isoform and, putatively, for a second mitochondrial isoform, which contains the canonical N-terminal domain and which is able to aminoacylates only the canonical $tRNA^{Thr}$.

Supp. Figure S3

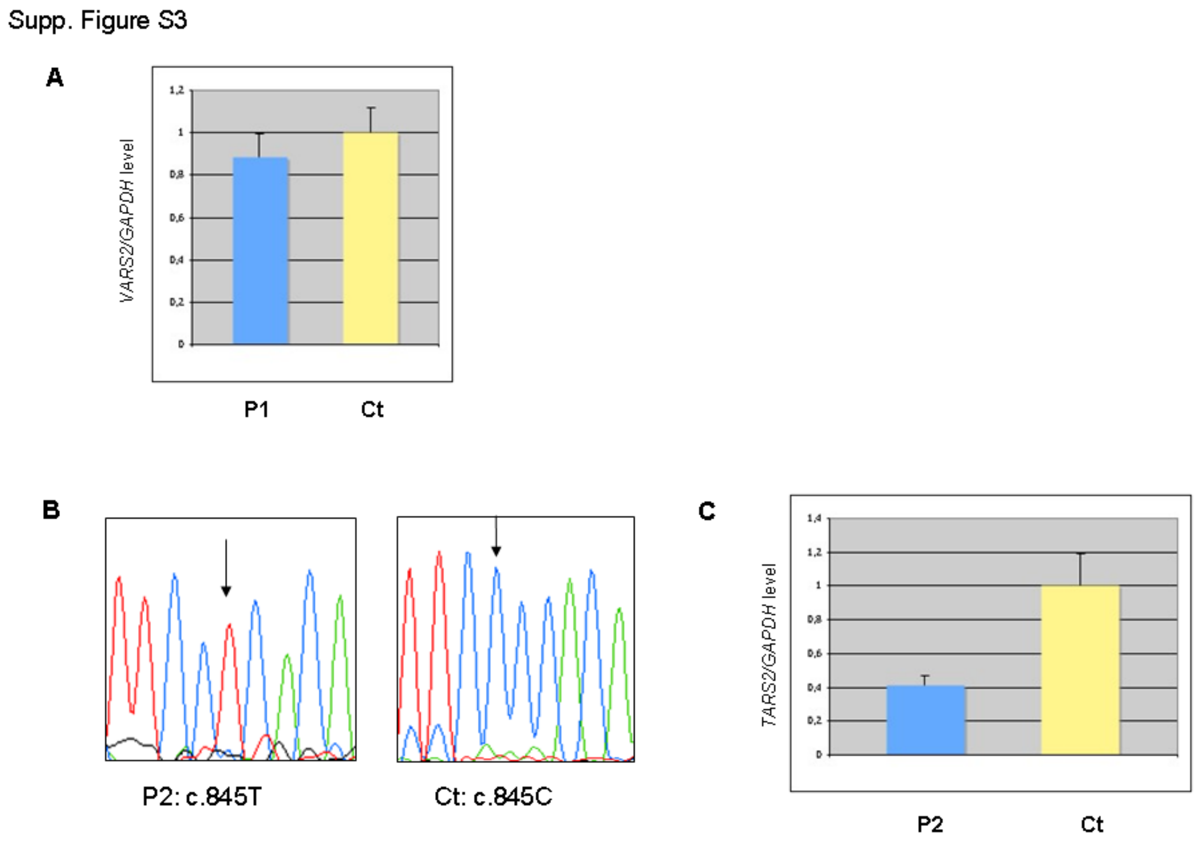

Analysis of *VARS2* and *TARS2* transcripts

A. Levels of *VARS2* transcript, normalized to that of the endogenous *GAPDH* cDNA, in fibroblasts from patient 1 (P1) and a control (Ct), obtained from 2 independent experiments, performed in triplicate.

B. Electropherograms of *TARS2* cDNA portion containing the c.845C>T variant, in patient 2 (P2) and a control subject (Ct).

C. Levels of *TARS2* transcript, normalized to that of the endogenous *GAPDH* cDNA, in fibroblasts from patient 2 (P2) and a control (Ct), obtained from 2 independent experiments, perfomed in triplicate.

Supp. Figure S4

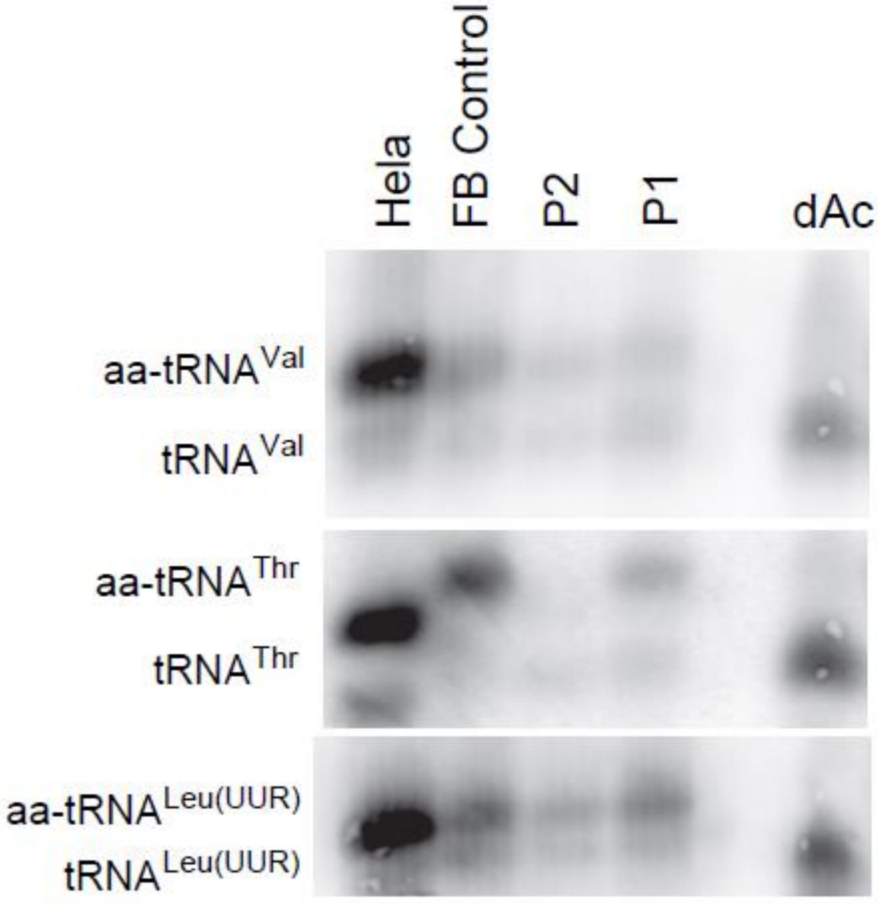

Aminoacylation of mitochondrial tRNAs

Mitochondrial tRNA aminoacylation analysis in RNA samples from patient 1 (P1), patient 2 (P2) and control fibroblasts (FB) using Northen blotting as described in the methods section. Membranes were hybridized with radioactive tRNA probes as indicated. “dAc” indicates deacylated control sample.

Supp. Figure S5

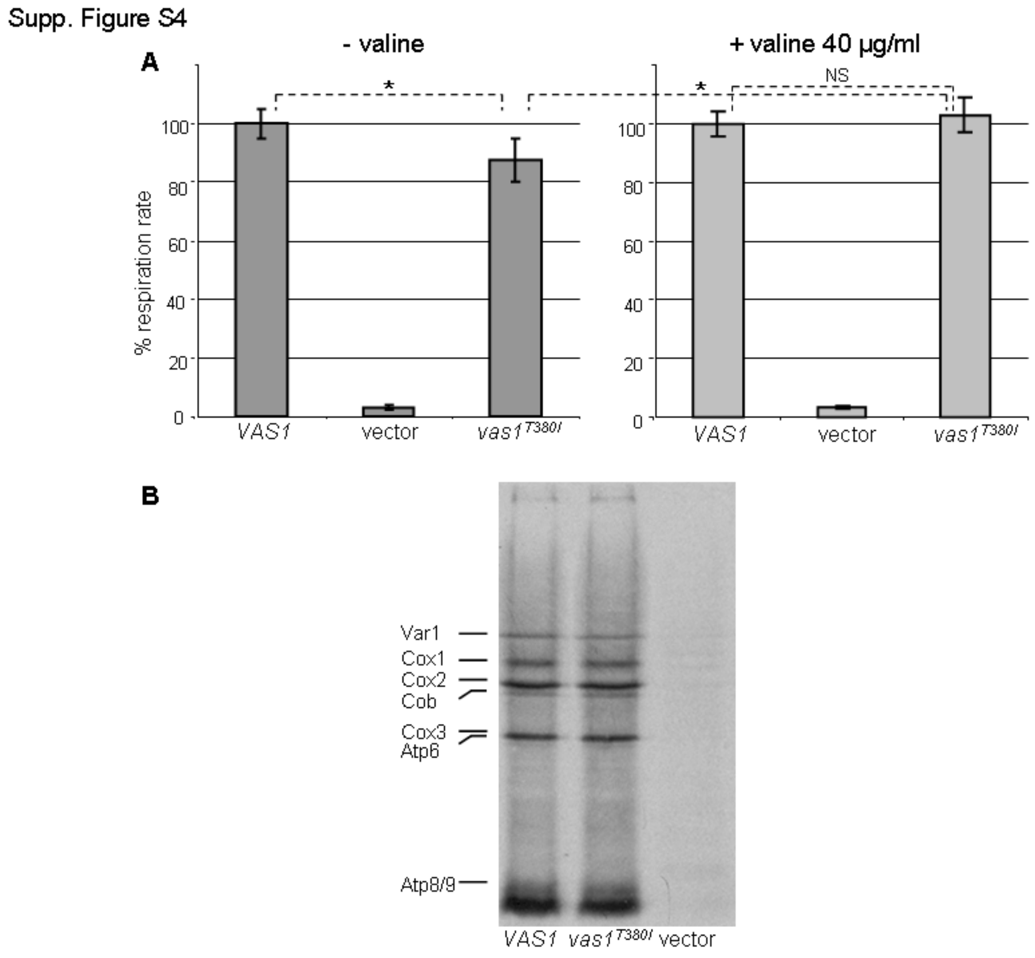

Yeast studies

A. Respiratory activity of wild type and mutant *vas1* yeast strains. Respiratory activity was measured after 18-hour growth in SD medium supplemented with 0.6% glucose, without valine (left panel) or with the addition of 40 µg/ml valine (right panel). Values are means of four independent experiments. *=$p<0.05$ in a two-tailed, unpaired t test.

B. *In vivo* mtDNA-dependent protein synthesis in yeast strains. Synthesis was performed after growth in SC medium supplemented with 2% galactose and 0.2% glucose when $OD_{600}\cong 1.5$.

Supp. Table S1. Primers used for *VARS2* and *TARS2* PCR amplification

Assay	Gene symbol	Forward primer	Reverse primer	Exon
**PCR (genomic DNA)**	*VARS2*	TTTCCAGTTCTACTGCCTTTAGC	GCCACCATTTAAAACCCAAG	10
	TARS2	TGGCTCTTCCAGGACATCTT	CCATTTCTTTTTCTGGAGCA	6
	TARS2	TCATTTGAGTCTTGAAAAAGGTG	GCTCCCAATTTCATAATCAGG	8
**Assay**	**Gene symbol**	**Forward primer**	**Reverse primer**	
**qPCR (cDNA)**	*VARS2*	ACAGCCCCCGATATGTTGAG	GGCCTGATATTCTGGTTTGAAGA	
	VARS2	GACCTTGCTCGTTTCTACCC	ATGAAGAAGCACCTTGCTGA	
	TARS2	AGGTGGCGGTGTCTCCG	ACTGCCGTGTGTAGCCTGC	
	TARS2	GGCCGAACAGGTCCTTAAACA	CAGAGTTGAGGTCCCAGGGTT	
	GAPDH	CTCTGCTCCTCCTGTTCGAC	ACGACCAAATCCGTTGACTC	

Supp. Table S2. Oligonucleotides used for the yeast manipulation

	**Primer forward**	**Primer Reverse**
**Cloning**	cgggggagctcgacctatgacatttctctcacag	ccgccctgcagcacctcgtctagttgtatagc
**Disruption**	gttgttgtatgtccttcagatttatggaattacttagatggt gcttcgtacgctgcaggtcgacg	tattacattaatttttatctaccttcaatctacaatttcaaaga tatcatcgatgaattcgagc
**Mutagenesis Cytvas1** [a]	ggaattacttagatggtGCgaataagtggttaaacaca ttatctaag	cttagataatgtgtttaaccacttattcGCaccatctaagt aattcc
**Mutagenesis vas1**^{T380I} [a]	cgatgaaaaactgatcattgctacaaTtagacctgaaa ctatatttgg	ccaaatatagtttcaggtctaAttgtagcaatgatcagtttt tcatcg

[a] In upper case the bases which have been changed to introduce the desired mutations

Supp. Table S3. In silico prediction of pathogenicity for the identified *VARS2* and *TARS2* mutations

	**Mutation taster**	**Polyphen2[a]**	**PMUT[a]**	**Panther[a]**
VARS2 c.1100C>T, p.Thr367Ile	Disease causing	Damaging (0.998)	Pathological (0.606)	Intermediate (0.45)
TARS2 c.845C>T, p.Pro282Leu	Disease causing	Damaging (0.974)	Neutral (0.254)	Probably deleterious (0.83)
TARS2 c.695+3A>G	Disease causing	/	/	/

[a] In parentheses the scores of pathogenicity (ranging from 0=benign to 1=highly damaging) obtained with the different softwares.

Mutation taster: http://www.mutationtaster.org;

Polyphen2: http://genetics.bwh.harvard.edu/pph2/index.shtml;

PMUT: http://mmb.pcb.ub.es/PMut;

Panther: http://www.pantherdb.org/tools/csnpScoreForm.jsp
